# FOLFOX activity in a rare case of metastatic colonic adenocarcinoma of the tongue: a case report

**DOI:** 10.1186/s12885-018-4395-5

**Published:** 2018-04-26

**Authors:** Clizia Zichi, Marco Tampellini, Marcello Tucci, Cristina Sonetto, Chiara Baratelli, Maria P. Brizzi, Giorgio V. Scagliotti

**Affiliations:** Medical Oncology, Department of Oncology, University of Turin, Azienda Ospedaliera Universitaria San Luigi, Regione Gonzole 10, 10043 Orbassano, Turin, Italy

**Keywords:** Oral cavity tumor, Colonic adenocarcinoma of the tongue, Chemotherapy, Metastatic disease, Folfox

## Abstract

**Background:**

Adenocarcinomas of the oral cavity are rare neoplasms, and only four cases of primary colonic adenocarcinoma of the tongue have ever been described in literature. Very few information about chemotherapy sensitiveness of this type of neoplasia is available, with only one regimen that showed some activity in a metastatic patient.

**Case presentation:**

We describe the case of a patient bearing a metastatic colonic adenocarcinoma of the tongue submitted to a first-line chemotherapy with oxaliplatin, 5-fluorouracil and folinic acid (FOLFOX regimen). After chemotherapy the patient obtained the complete disappearance of the primitive neoplasia located in the body of the tongue, and a tumor size reduction > 50% of liver and lung metastases.

**Conclusions:**

This case demonstrated the activity of the combination of oxaliplatin and 5-fluorouracil in this very rare neoplasia. The FOLFOX regimen might be considered either in advanced and especially in the neoadjuvant setting, when the reduction of the primary tumor is highly needed.

## Background

In Western countries tumors of the oral cavity are rare and account for four new cases every 1,000,000 persons/year in Italy [1]. These tumors usually develop from precancerous lesions such as leukoplakia, erythroplakia, sub mucosal fibrosis and lichen, or from continuous mucosal inflammation due to irregular tooth placement or damaged dental prosthesis. However, in many cases the specific pathogenesis remains unknown. Tongue is the most common site among Caucasians (40–50% of oral cancers). They are typically squamous cell carcinomas (95%), whereas adenocarcinomas, usually originating from minor salivary glands, are rare and mainly reported in women and younger patients [2]. Similarly, primary colonic adenocarcinomas of the tongue (CAT) are extremely uncommon, and only four cases have been so far described [3–5], all of them with localized disease at diagnosis.

The case of a patient with an intestinal adenocarcinoma of the body of the tongue with synchronous distant metastases responsive to FOLFOX regimen is here reported.

## Case presentation

**Fig. 1:**
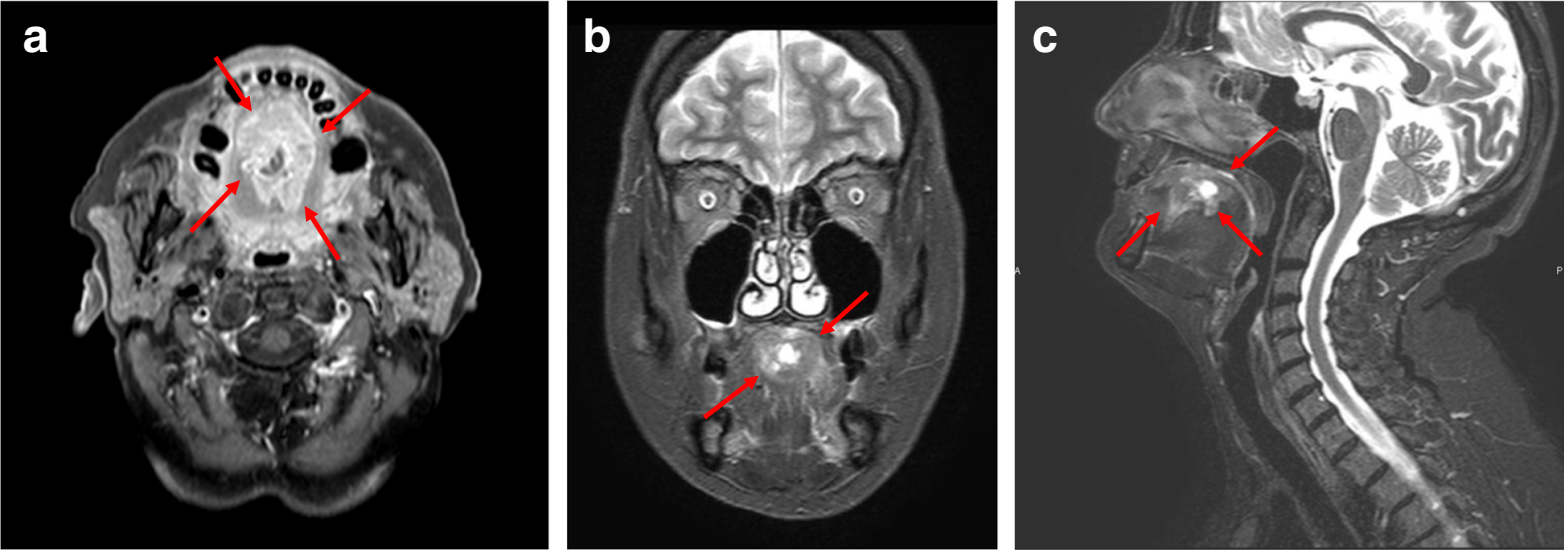
RMN scan of the oral cavity with a bulky, solid, infiltrating lesion of 47x27x22 mm of the body of the tongue, with central loss of substance and irregular hyper-vascularity. Transversal (**a**), coronal (**b**), and sagittal (**c**) view

**Fig. 2:**
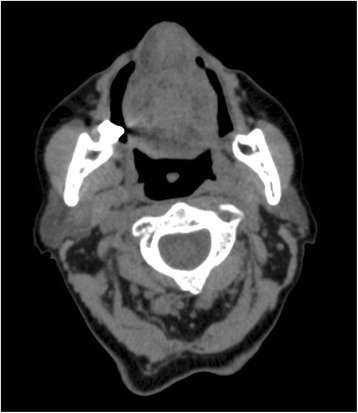
CT scan after 6 months of chemotherapy showing the complete disappearance of the tumor mass primarily located in the body of the tongue

In June 2013, a 75-year-old woman was diagnosed with a solid mass located in the body of the tongue (Fig. 1) with suspected lateral cervical and submandibular lymphnodes. A biopsy of the tongue revealed an adenocarcinoma forming invasive glandular structures with areas of luminal necrosis. Immunohistochemical stainings yielded positivity for CK20 and CDX2, and negativity for CK7, findings compatible with a colonic adenocarcinoma subtype. Computerized tomography (CT) with iodine contrast revealed multiple lung metastases and a large hepatic lesion of 83 × 60 mm. The patient was subsequently submitted to gastroscopy and colonoscopy, both negative for neoplastic disease. From September 2013 to March 2014 the patient received 12 cycles of FOLFOX regimen. Treatment was generally well tolerated with improvement of patient quality of life, reduction of dysphagia and recovery of a normal eating. Body weight increased and local pain control was satisfactory. The CT scan performed in April 2014, at the end of the chemotherapy program (Fig. 2), showed the complete response of the primary lesion, together with the partial response of the lymphadenopathies and lung metastases. Liver metastases presented conflicting findings because the large secondary lesion decreased in size (40 mm), but new satellite lesions were detected. Additionally, a vertebral metastasis was clearly visible in the body of L4 and a compound fracture was evident in the left ischio-pubic branch. The patient was hospitalized in May 2014 for uncontrolled pain. General condition progressively worsened preventing any further treatment and finally she died in October 2014, 16 months after diagnosis.

## Discussion and conclusions

This is the first reported case of a primary colonic adenocarcinoma of the tongue with synchronous metastases, the first appeared in a woman and the fifth ever described.

The histogenesis of this cancer is uncertain, but the neoplastic transformation of a minor salivary duct epithelium is quite likely. The hypothesis is supported by the immunohistochemical profile of the tumor and by studies of primary intestinal-type adenocarcinoma of the sino-nasal tract where evidence of respiratory epithelium transformation to an intestinal phenotype have been noted in the adjacent non-neoplastic epithelium [6]. In addition, the possibility that these tumors derived from a gastrointestinal tract heterotopia within the tongue has been proposed [7]. The tumor we reported was histologically similar to those of previous reports, with invasive malignant glandular structures and no involvement of the overlying squamous mucosa.

The mainstay of treatment of adenocarcinoma of the oral cavity is surgery. Thus, neoadjuvant treatments may result in better outcomes. However, the lack of knowledge due to the rarity of these neoplasms hampered the definition of specific guidelines.

Differently from what we observed in our patient, all the reported cases presented at diagnosis with locoregional lymphnodal metastases and no distant metastases. Two patients [3] received unspecified and inefficacious neo-adjuvant chemotherapy and were alive without relapse of the disease 11 and 13 months after surgery. One case [4] underwent surgery followed by adjuvant radiotherapy without chemotherapy. This latter case was published when radiotherapy was ongoing and thus no valid follow-up information was provided. The last case [5] received carboplatin and taxol with radiotherapy as post-resection adjuvant therapy. Patient relapsed into the lung 14 months after diagnosis and then received permetrexed and bevacizumab therapy with an overall survival of 60 months.

Chemotherapy of adenocarcinoma of the oral cavity is very heterogeneous and includes cisplatin, carboplatin, paclitaxel, 5FU, anthracyclines, gemcitabine, and cyclophosphamide, within others [8]. Little information about systemic therapy of CAT is available. Due to the uncertain outcome of the previous clinical reports we decided to administer 5FU and oxaliplatin (FOLFOX schema). This therapeutic decision was made because the more extensively studied drugs in adenocarcinomas of the oral cavity are platinum derivatives (cisplatin and carboplatin) [8] and the combination of platinum with 5FU normally results in a synergistic activity [9]. In addition the combination of oxaliplatin and 5FU is active against either squamous cell carcinomas of the oral cavity and adenocarcinomas of other origins including colorectal.

Chemotherapy was well tolerated with an immediate clinical benefit; particularly dysphagia that early improved allowing the patient a regular eating. Chemotherapy induced a good local control of the disease, with significant tumor shrinkage at the end of therapy, and a decrease in size of liver and lung metastases. These results encourage clinicians to consider FOLFOX regimen as neoadjuvant treatment of CAT. It should be underlined that oxaliplatin and 5FU might also be associated to radiotherapy, expanding the possibility of treatment in locally advanced tumors.

Overall survival of patients bearing salivary gland carcinomas is heterogeneous, with 5-year survival of nearly 50% in high-grade tumors. Palliative chemotherapy did not demonstrated to be highly active, with response rate ranging from 20 to 25% down to 10% according to histological subtype, type of chemotherapy and reported series [10]. The case we reported survived 16 months. Unfortunately patient quality of life was hampered by bone metastases and this clinical condition prevented any further line of chemotherapy administration. To the best of our knowledge, we report the first case of a response to chemotherapy of the primary tumor. FOLFOX regimen was well tolerated and showed high activity and then should be considered when planning an induction chemotherapy or chemo-radiotherapy prior to surgery in locally advanced disease.

## Abbreviations

CAT: Colonic adenocarcinomas of the tongue
CT: Computerized tomography
FOLFOX: Folinic acid, 5fluorouracil, oxaliplatin regimen
